# Design Optimization of a Pneumatic Soft Robotic Actuator Using Model-Based Optimization and Deep Reinforcement Learning

**DOI:** 10.3389/frobt.2021.639102

**Published:** 2021-05-07

**Authors:** Mahsa Raeisinezhad, Nicholas Pagliocca, Behrad Koohbor, Mitja Trkov

**Affiliations:** Department of Mechanical Engineering, Rowan University, Glassboro, NJ, United States

**Keywords:** soft robotics, soft actuators, design optimization, design of soft robots, firefly algorithm, deep reinforcement learning

## Abstract

We present two frameworks for design optimization of a multi-chamber pneumatic-driven soft actuator to optimize its mechanical performance. The design goal is to achieve maximal horizontal motion of the top surface of the actuator with a minimum effect on its vertical motion. The parametric shape and layout of air chambers are optimized individually with the firefly algorithm and a deep reinforcement learning approach using both a model-based formulation and finite element analysis. The presented modeling approach extends the analytical formulations for tapered and thickened cantilever beams connected in a structure with virtual spring elements. The deep reinforcement learning-based approach is combined with both the model- and finite element-based environments to fully explore the design space and for comparison and cross-validation purposes. The two-chamber soft actuator was specifically designed to be integrated as a modular element into a soft robotic pad system used for pressure injury prevention, where local control of planar displacements can be advantageous to mitigate the risk of pressure injuries and blisters by minimizing shear forces at the skin-pad contact. A comparison of the results shows that designs achieved using the deep reinforcement based approach best decouples the horizontal and vertical motions, while producing the necessary displacement for the intended application. The results from optimizations were compared computationally and experimentally to the empirically obtained design in the existing literature to validate the optimized design and methodology.

## 1. Introduction

Recent developments in soft robotic technologies has enabled a fundamental shift and advancement in robots abilities (Laschi et al., 2016) and shifted paradigms in the domain of human-machine-environment interactions. Inherent softness and compliance of soft robots provides advantages over traditional rigid-body robots and actuators due to their unique capabilities to conform, comply, and safely interact with uncertain and dynamic environments (Laschi, 2015). Utilizing these advantages, soft robotic technologies have been successfully deployed in several applications including manufacturing, search and rescue explorations, and biomedical and rehabilitation engineering (Wang and Lida, 2015; Galloway et al., 2016; Hines et al., 2017; Walsh, 2018). However, despite recent advancements achieved in the areas of functional materials and design approaches, soft robot designs often rely on engineering intuition or are bio-inspired (Kim et al., 2013). Systematic approaches for the design and development of soft structures and actuators are needed to further extend the capabilities and functionality of soft robots and exploit their advantages to achieve their full potential.

Many existing soft actuators are designed as fluid-driven elastic inflatable structures with their anisotropic flexible body serving as a host to a controlled pressurized fluid as the actuation input (Gorissen et al., 2017). The fluid chamber layout, shape, and size, along with the material composition and overall structure design all determine the functionality and capabilities of these soft machines. Soft robots can exhibit one primary mode of actuation being either linear extensile and contractual, axial and helical torsional, or planar and non-planar flexural bending motions (Marchese et al., 2015; Gorissen et al., 2017).

The existing literature has investigated various model-based designs of pneumatically driven soft actuators. In Garriga-Casanovas et al. (2018), a framework for the design of soft robotic manipulators with fluidic actuation was presented for bending, extending, and contracting devices considering force equilibrium in the fluid chamber. Sipos and Peter (2020) used a model-based approach coupled with a root finder algorithm for optimization of maximum displacement. Recently, Boyer et al. (2020) utilized beam modeling in soft robotics via parameterization of stain-fields, and Jiang et al. (2019) used a tapered beam approach to explore the use of a chain like structure for stiffness regulation in a soft-rigid manipulator. Most of the above-mentioned approaches require numerical methods and advanced analysis techniques, which may be cumbersome for practical design of soft systems. Furthermore, structural modeling of simple interconnected beams has not been considered previously in soft robotic modeling approaches and offers advantages due to its simplicity.

The integrated structure of non-articulated soft actuators hinders the independent control of multi-degree-of-freedom (DOF) motion, due to its degrees of freedom being coupled. Soft actuators capable of generating independent horizontal and vertical motions can be used for controlled motion planning or contact surface manipulation in human-machine interactions applications to precisely and independently control their motion and contact loads. Successful surface manipulations using shape morphing soft actuators that can generate rolling, wrapping, and saddle like motions have been recently demonstrated using a soft robotic pad (Sun et al., 2017, 2020) and fibers with voltage-actuated dielectric elastomer beams (Shain et al., 2015). A soft actuator for surface manipulation developed in Raeisinezhad et al. (2020) could partially achieve two-axis translational motions independently, however, its design was obtained empirically without exploring its full potential by applying a rigorous design optimization approach.

Many optimization methods have been used to optimize fluid chamber designs of soft actuators for various applications. Dammer et al. (2018) used finite element modeling with gradient-based optimization for shape optimization of bellows actuators. Skouras et al. (2012) considered optimization of inflatable structures using augmented Lagrangian methods. Design optimization of a six chambered soft robot in Guo and Kang (2020) was performed using the non-linear programming by quadratic Lagrangian (NLPQL) coupled with Finite Element (FE) modeling. A genetic algorithm was utilized in the multi-objective design optimization of a soft multi-DOF manipulator (Bodily, 2017) and in the design optimization of a soft pneumatic actuator (Runge et al., 2017). In design optimizations, the algorithms that can handle non-linear and high dimensionality problems are favorable, due to complex multi-parameter structure-material relations present in soft robotic systems. Swarm intelligence presents a promising solution to such problems (Gandomi et al., 2015). A case study performance comparison of swarm methods in a cantilever structural optimization, namely accelerated particle swarm optimization, a classical particle swarm optimization, cuckoo search, and firefly algorithm, demonstrated that the firefly is an effective optimizer in model-based structural optimization (Gandomi et al., 2015). Furthermore, comparative work on the effectiveness of swarm intelligence methods vs. evolutionary methods have shown that swarm intelligence based methods are more accurate and robust than evolutionary algorithms (Kurban et al., 2014).

The rapid progress of machine learning methods in recent years has demonstrated that these algorithms can be applied for design problems. Deep reinforcement learning (DRL) in particular, is well-suited for handling complex non-linear environments for both design and control, however, limited work exists on using DRL for static design optimization. Among those, in Viquerat et al. (2021), proximal policy optimization (PPO) was used in shape optimization, where indirect supervision from a generic reward signal is used as a non-linear optimizer. Deep Q-Network (DQN) was shown to be successful in optimizing the design of the angle of attack of airfoils (Yonekura and Hattori, 2019) and similarly, double-DQN with hindsight experience replay (HER) demonstrated good performance in design optimization of microfluidic devices for flow sculpting (Lee et al., 2019). However, utilizing machine learning for soft robot design has not been fully explored and our work complements the existing research in this field.

Topology optimization has been previously employed for various design objectives in soft robotics with FE solvers utilizing the software-in-a-loop design optimizations. In de Souza and Silva (2020), a density based topology optimization was used to maximize displacement of a pressure driven actuator, while constraining the material's volume. The interior point algorithm (IPOPT) was used to solve the optimization problem. Caasenbrood et al. (2020) presented a topology optimization scheme to find the optimal design of a pressure-driven soft robots structure using the Solid Isotropic Material Method with Penalization (SIMP) in which they assign a continuous density variable for each element. Zhang et al. (2018) provided a method for topology optimization of a pneumatically actuated soft gripper by recasting the design problem mathematically and using Solid Isotropic Material with Penalization method which uses densities of the discretized elements as design variables. The above-mentioned methods focus primarily on optimizing the weight-strength ratio and do not directly employ advanced optimization techniques, such as DRL. Our work complements the existing software-in-a-loop design optimizations by combining the FE solver (i.e., pyANSYS) and a deep reinforcement learning approach.

The design of soft robots and actuators has many applications in human-machine interactions and biomedical applications, due to their unique softness and safety. A specific example demonstrating the use of a soft actuator for pressure injury prevention was proposed in Raeisinezhad et al. (2020). The existing literature on pressure injury prevention have proposed devices for regulating normal loads at the human-machine interface (Fiedler et al., 2009), although it is well-known fact that the physical tissue damage is caused by the repetitive contact friction loads that cause shear stress on skin, which are the most important factor in blister formation (Polliack and Scheinberg, 2006). However, due to the physical complexity of blister and pressure injury prevention devices, only a few of them have capabilities to prevent shear loading. Eilbeigi et al. (2017) proposed a soft-structured bed with integrated sensors that can control the force applied to its surface by automating the tasks required for preventing pressure injuries of disabled people. A rare examples of an existing pressure injury prevention system that considers shear as well as normal stresses include a soft air bladders system that can control the local orientation of the bladder reported in Yousefi et al. (2011), and a similar system that utilizes multi-pins attached to small air bladders underneath to evenly distribute the skin-pin interaction loads (Fiedler et al., 2009). Using systematic modeling and optimization approaches to develop a soft actuator with decoupled degrees of freedom can ameliorate issues in pressure injuries and blister formation at the human-machine interface that many existing works have largely neglected.

The main contribution of this work lies in the formulation and creation of a deep reinforcement learning design optimization framework using an analytical and finite element environment to improve the mechanical performance of soft robots. In addition, we propose a multi-beam model-based design optimization scheme, where we extend an analytical modeling approach considering tapered and thickened beams to form a multi-chamber structure acting as a soft actuator. Direct performance comparison of firefly- and DRL-based optimizations is provided and discussed. We quantify the efficacy of the analytical model-based optimization compared to the FE computational framework that has an increased variable design space. We specifically demonstrate the developed framework for the design of an individual soft actuator to be used in a multi-actuator soft robotic pad for the application of blister and pressure injury prevention as proposed in Raeisinezhad et al. (2020).

## 2. Modeling

### 2.1. Analytical Model

**Figure 1:**
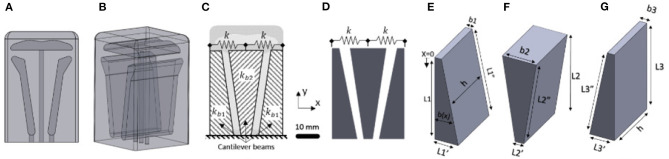
3D model of the soft robotic actuator in **(A)** front view and **(B)** orthogonal view. **(C)** Model of the actuator specifying the naming convention for the cantilevered beams. **(D)** Schematic of cantilever model of the actuator considering the spring in between. **(E)** Schematic of the first cantilever model of the actuator. **(F)** Schematic of second cantilever model of the actuator. **(G)** Schematic of third cantilever model of the actuator.

We consider the design optimization of a soft actuator proposed in a previous work (Raeisinezhad et al., 2020), see Figures 1A,B. The pneumatic actuator can achieve near independent vertical and one-axis horizontal motions using pressurized air in its three air chambers, while simultaneously withstanding normal pressures of 20 kPa applied at the top surface. The actuator was designed empirically and herein, we focus on design optimization of the air chambers geometry to produce maximal horizontal motion.

We reduce our model from a 3D representation into a 2D problem and consider only the cross-section. We assume symmetry about the x-y plane shown in Figure 1 and model our system at the central cross section. The top chamber shown in Figures 1A–C is neglected to independently analyze only the horizontal motion. The actuator is modeled as a system of three cantilever beams with linearly varying rectangular cross sections (Figures 1C–G), interconnected by virtual spring elements (Figures 1C,D). The modeling approach was inspired based on the empirically obtained design from Raeisinezhad et al. (2020). The actuator is also assumed to be symmetric about the y-z plane about the center of the middle cantilever shown in Figure 1, establishing both beams in Figures 1E,G have the same geometric parameters.

Cantilever beams with varying rectangular cross sections are modeled using Euler-Bernoulli beam theory. We extend the analytical formulation for the beam deflection of both tapered (Romano and Zingtone, 1992) and thickened beams to derive an analytical model for this system, by connecting beams with spring elements and considering principle of superposition. This method of modeling was selected as a basis for shape and geometry optimization of the air chambers. We define the curvature of the beam considering distributed load as

(1) $$\begin{array}{r} \frac{d^{2}}{dx^{2}}\left(EI\left(x\right)\frac{d\phi }{dx}\right)=q\left(x\right), \end{array}$$

where *E* is the Young's modulus, *I*_*zz*_(*x*) is the moment of inertia around the *z*-axis, *q*(*x*) is the distributed load applied on each cantilever beam, and ϕ is the slope of the cross section defined as $\frac{dy}{dx}=\phi$ considering the Euler-Bernoulli beam theory. The general solution of tapered beam deflection is given by

(2) $$\begin{array}{l} y_{i}\left(x\right)=C_{1}+C_{2}ln\left(\alpha_{i}x+b_{i}\right)+C_{3}\left(\alpha_{i}x+b_{i}\right)+\frac{C_{4}}{\left(\alpha_{i}x+b_{i}\right)}\\ \;+\frac{3q_{i}}{\left(2\alpha_{i}^{4}Eh\right)}ln\left(\alpha_{i}x+b_{i}\right)\left(\alpha_{i}x+b_{i}\right) \end{array}$$

where α_*i*_ is the slope, *q*_*i*_ is the applied distributed load for given pressure/vacuum in the air chambers, *E* is elastic modulus of the material (defined later in this section), *h* is width of the air chamber, and *b*_*i*_ is the thickness of the tapered and thickened cantilever beams respectively at *x* = 0, for *i*−*th* beam (*i* = 1, 2, 3). We solve (2) using two boundary conditions at the tip (*x* = 0) and two at the fixed end (*x* = *L*) of the cantilever beam to obtain coefficients *C*_1_, *C*_2_, *C*_3_, and *C*_4_.

Using the principle of superposition we solve for the equilibrium of displacements due to both distributed loading along the length of the beam (i.e., internal pressure/vacuum inside chambers) and concentrated loading from a virtual spring at the free tip of the beam. The virtual spring resembles the functionality of the thin elastic material forming a closed chamber. Solving for the overall deflections of all three beams (*Y*_*o*_1__, *Y*_*o*_2__, and *Y*_*o*_3__) we obtain

(3) $$\begin{array}{r} Y_{o_{1}}=\frac{y_{1}+Y_{o_{2}}\left(\frac{k_{s}}{k_{b_{1}}}\right)}{1+\frac{k_{s}}{k_{b_{1}}}}, \end{array}$$

(4) $$\begin{array}{r} Y_{o_{2}}=\frac{y_{2}+y_{1}\frac{\frac{k_{s}}{k_{b_{2}}}}{1+\frac{k_{s}}{k_{b_{1}}}}-y_{3}\frac{k_{s}k_{b_{3}}}{k_{b_{2}}\left(k_{b_{3}}+k_{s}\right)}}{\left(1-\frac{k_{s}^{2}k_{b_{3}}}{k_{b_{3}}k_{b_{2}}\left(k_{b_{3}}+k_{s}\right)}-\frac{k_{s}^{2}}{k_{b_{2}}\left(k_{b_{1}}+k_{s}\right)}+\frac{2k_{s}}{k_{b_{2}}}\right)}, \end{array}$$

(5) $$\begin{array}{r} Y_{o_{3}}=\frac{Y_{o_{2}}\left(\frac{k_{s}}{k_{b_{3}}}\right)-y_{3}}{1+\frac{k_{s}}{k_{b_{3}}}}, \end{array}$$

where *k*_*s*_ is the stiffness of the material between the cantilevers (virtual springs) and *k*_*b*_1__, *k*_*b*_2__, and *k*_*b*_3__ are the equivalent beam bending stiffness's associated with each cantilever beam. The relationships among deflections of all three beams are coupled through virtual spring stiffness *k*_*s*_ that depends on the material and the dimensions of the solid that connects the beam. We use this set of equations in two different optimization methods to determine optimal chamber geometries that lead to the maximal horizontal and minimal vertical motion. The optimization methods are discussed in the next section.

### 2.2. Computational and Constitutive Material Model With Experimental Material Characterization

We selected silicone rubber (Elite Double 22, Zhermack, Badia Polesine, Italy) as the material for the design and fabrication of the actuator. The material hardness is Shore A 22, similar to the one in Raeisinezhad et al. (2020). We characterize the material properties to be used as the input into FE computational models. We followed the ASTM D638 standard in preparation and testing of the custom molded material samples. A universal testing machine (Shimadzu AGS-X 10kN, Columbia, MD) and digital image correlation (Correlated Solutions, Irmo, SC) were used in the characterization process. In-plane deformation response was obtained from measurements of *x*− and *y*−axis strain components, with tensile load applied along *y*−axis. Considering strains less than 0.05, we approximate the Young's Modulus *E* as 404*KPa* for use in the analytical model.

**Figure 2:**
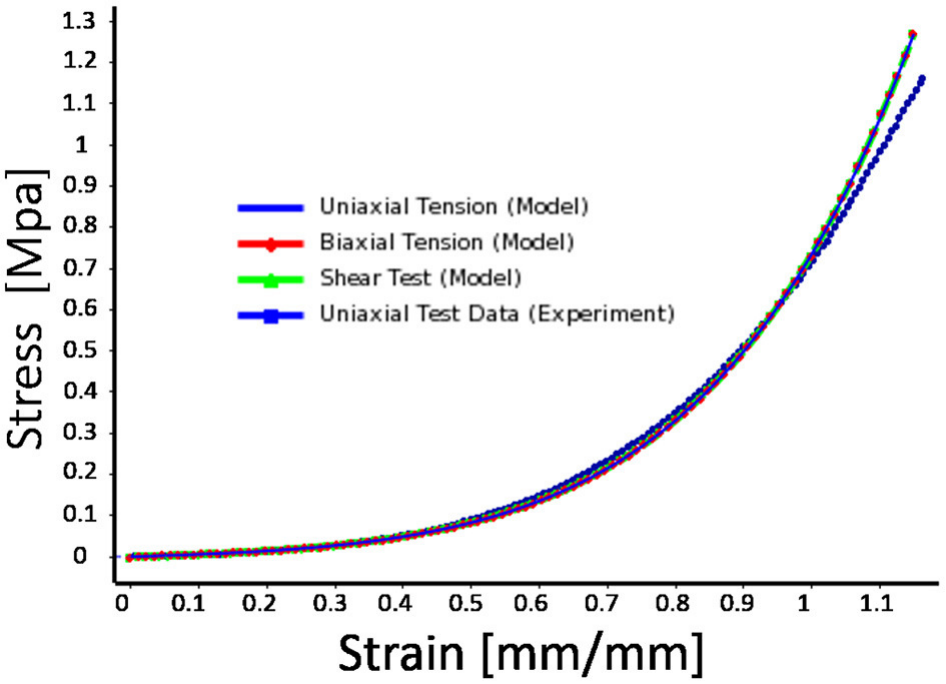
Experimental material characterization results with hyperelastic material model fit (first-order Ogden model). Strain range is greater than maximal principal strains of the actuator (< 1.0) at maximum applied pressures/vacuum in air chambers.

Figure 2 shows material characterization curves from uniaxial tensile testing of the silicone rubber material. The non-linear behavior of the hyperelastic material was modeled using an Ogden model (Ogden, 1972), described by the strain energy function $U\left(\lambda_{1},\lambda_{2},\lambda_{3}\right)=\overset{N}{\underset{i=1}{\sum }}\frac{2\mu_{i}}{\alpha_{i}^{2}}\left({\bar{\lambda }}_{1}^{\alpha_{i}}+{\bar{\lambda }}_{2}^{\alpha_{i}}+{\bar{\lambda }}_{3}^{\alpha_{i}}-3\right)+\overset{N}{\underset{i=1}{\sum }}\frac{1}{D_{i}}{\left(J_{e}-1\right)}^{2i}$, where μ_*i*_, α_*i*_, and *D*_*i*_ are the material parameters obtained from the sample tensile test data. The $\lambda_{k}^{\alpha_{i}}$ are the principal stretch ratios with the volume change removed, and *J*_*el*_ is the ratio between current and original volume excluding thermal effects. Assuming incompressibility of the silicone material, we use the first-order Ogden model to fit the data for the nominal stain until 1.2.

The material model coefficients were μ_1_ = 45, 480, α_1_ = 2.4914, and the incompressibility parameter *D*_1_ = 0. The parameters were input into the FE computational model using ANSYS™Workbench software. We model our actuators using higher order 20-node SOLID186 and higher order 10-node SOLID187 elements to support hyperelasticity and large strains in the model, and consider contact interactions between the cantilevers. In the simulation, each actuator was fixed at the base and pressure/vacuum was incrementally increased.

## 3. Optimization Methods

We performed three different optimization approaches using combinations of the analytical and finite element (FE) model with the firefly algorithm (FA) and deep reinforcement learning (DRL) optimization methods. The analytical model was used with both FA and DRL optimizations to cross-validate the optimization results. The FE model was used with the DRL to compare and validate the developed analytical model and further explore design space in search of optimal air chamber design parameters. The extended capabilities of the FE model allow for applying realistic loads and minimally constrained geometry that the analytical model cannot provide or capture in full capacity. In the following section, all three optimization approaches are presented.

### 3.1. Analytical Model and Firefly Algorithm

The firefly algorithm (FA) is a part of a class of swarm intelligence algorithms that has been shown to be effective in non-linear multi-modal optimization problems (Yang, 2010). The algorithm was shown to be suitable for solving structural engineering problems, including stepped cantilever beam design problems (Gandomi et al., 2011). We specifically chose a modified FA in our analysis containing memory functionality (Kazemzadeh-Parsi, 2014) that preserves the information about the best particle and global position and uses this information in the next iteration. Due to similarities between previously reported and our structural optimization process, we selected FA for design optimization of our soft actuator.

The soft actuator is optimized to achieve maximal horizontal displacement of the top surface. We formulate the optimization problem as a cost function that aims to maximize the deflection of the first (left) side beam, while minimizing the deflection of the second (middle) and third (side/right) beams from their midpoint. Minimizing the midpoint deflections is to prevent the overlap between beams, while still allowing contact between the sides of beams, when the pressure differential is applied. The problem is formulated as minimizing the cost function, while satisfying the geometric constraints and is expressed as:

(6) $$\begin{array}{l} \begin{array}{l} \text{minimize}-Y_{o_{1}}+Y_{o_{2mid}}+Y_{o_{3mid}}\\ \text{subject to }\left(Y_{o_{1}}-b_{1}\right)+\left(Y_{o_{2}}+\frac{b_{2}}{2}\right)-d_{1}>0\\ b_{1}+b_{3}+L_{2}^{\prime }-0.03>0\\ b_{1}+b_{2}+b_{3}+d_{1}-0.0508<0\\ L_{1}^{\prime }+L_{2}^{\prime }+L_{3}^{\prime }+d_{1}^{\prime }-0.0508<0\\ 0.1<\frac{L_{2}^{\prime }}{b_{2}}<0.9, \end{array}\\ \begin{array}{c} 0.1<\frac{b_{1}}{L_{1}^{\prime }}<0.9,\\ 0.1<\frac{b_{3}}{L_{3}^{\prime }}<0.9 \end{array} \end{array}$$

The design space includes four discrete parameters that define the shape of two beams (Figures 1E,G), due to the symmetry assumption. Imposed hard constraints are defined as $0.006m<L_{1}^{\prime }<0.025m$, 0.004*m* < *b*_1_ < 0.02*m*, $0.006m<L_{2}^{\prime }<0.025m$, 0.004*m* < *b*_2_ < 0.025*m*. We consider fixed height of the actuator *L*_1_ = 0.063*m* and fixed width of the air chambers *h* = 0.044*m*. We transform our constrained optimization problem into a series of unconstrained optimization problems using Powell's method, where the inequality constraints are enforced with a penalty function (Berhe, 2012).

We implemented a custom firefly algorithm using MATLAB (R2020a, MathWorks, Natick, MA) that builds on the code provided in Yang (2021). In the optimization, we limit the number of iterations to 50 and utilize 25 fireflies (i.e., swarm size), with an attraction coefficient base value (β_0_ = 2), a light absorption coefficient (γ = 1), a mutation coefficient (α = 0.2), a intensification factor (*q* = 0.5), and a mutation coefficient damping ratio (α_*damp*_ = 0.98).

### 3.2. Analytical Model and Deep Reinforcement Learning

**Figure 3:**
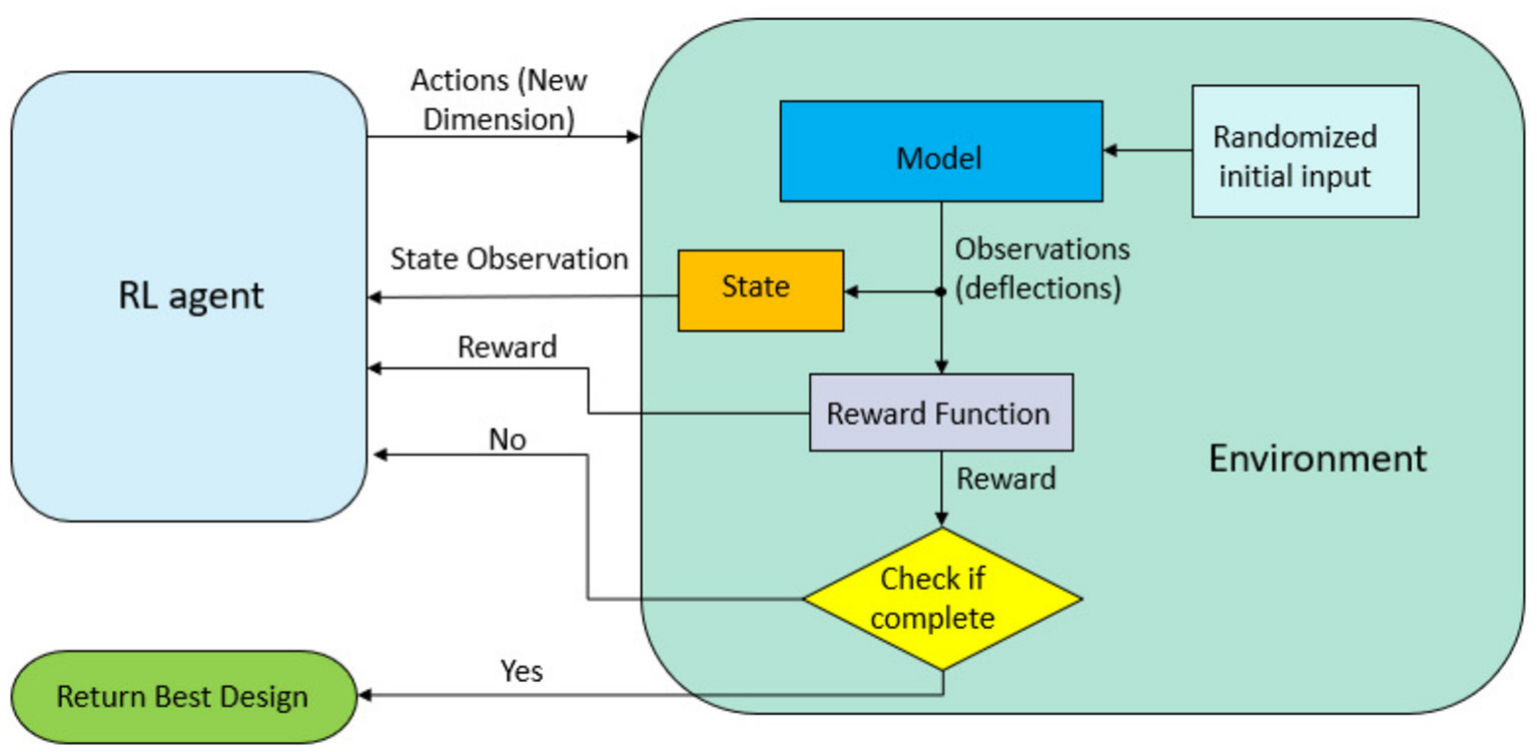
Schematics of the interactions between the RL agent and the environment. Each episode consists of one step. Geometry parameters that dictate the chamber layout are passed from the RL agent as actions. The state observation and a reward signal for indirect supervision are sent back to the RL agent.

We used a deep reinforcement learning approach in combination with our analytical model as the environment. Figure 3 shows the schematics of our DRL architecture. An agent interacts with the environment with the goal to perform the best action at a particular state, and is rewarded in accordance with the performed action (Sewak, 2019). We utilize the deep deterministic policy gradient method (DDPG) (Lillicrap et al., 2016) for our DRL-based optimization. It is a model-free, off-policy, actor-critic based algorithm. One of the key advantages compared to the other DRL algorithms used for optimization (i.e., Double DQN, Hasselt et al., 2016) is its ability to handle continuous action spaces. We adopt a single time-step episode methodology (Viquerat et al., 2021), where learning occurs from indirect supervision from a reward signal. Each episode, the reward is fed to the RL agent and used to determine how to maximize the reward. A total of 500 episodes were used with a batch size of 16. We considered different initial geometries and ran the model five times to check the convergence. We implemented the optimization algorithm using the Stable Baselines repository (Hill et al., 2018) to allow the method be easily adopted by a wider audience.

### 3.3. FE Computational Model for DRL Optimization

For the purpose of validating performance of our analytical model, we developed a finite element model of the proposed soft actuator and optimized it using the DRL-based DDPG optimization method. We utilized an ANSYS FE solver using the PyANSYS module (Kaszynski, 2020) to create a 2D finite element model considering only the cross-section of the actuator. We considered symmetry of air chambers to reduce the number of optimized parameters and guarantee functionality of symmetrical horizontal displacement of the actuator in both directions. A fixed boundary condition is used at the base of the actuator with pressure applied at the inner chamber surfaces that are same across all the other optimization routines. The static-structural optimization routine is used in the study.

The air chamber was defined using four ordered pairs of *x*− and *y*−coordinates that correspond to the vertices of the air chambers in the actuator. We use the actions from the RL agent as the coordinates of those vertices (i.e., key points). The ordered pairs are defined as (a_1_*x*__,a_1_*y*__), (a_2_*x*__,a_2_*y*__), (a_3_*x*__,a_3_*y*__), (a_4_*x*__,a_4_*y*__). We considered two variants of the FE model. In the first variation, we only controlled the *x*− coordinates of the ordered pairs (total 4 actions), while keeping *y*− coordinates fixed in similar location as those in the analytical model. Imposing these similarities between analytical model and FE environments allows a comparison and validation of our analytical model, in particular for the case when using same DRL optimization method. In the second variation, the *y*−coordinates were unconstrained and were part of the optimization variables in our FE model (total 8 actions). In both variations, we impose the same parameter constraints as that of the analytical model. The domain for each *x*− coordinate was defined as: 0.006*m* < a_1_*x*__ < 0.02*m*, a_1_*x*__ < a_2_*x*__ < 0.022 *m*, 0.015 *m* < a_3_*x*__ < 0.023 *m*, 0.004 *m* < a_4_*x*__ < a_3_*x*__. The line connections between key points form the chamber contour and are connected in a counter clockwise direction to avoid overlap. Considering the pre-defined outer shape of the actuator we can create a 2D FE mesh of the cross-section. In each iteration a 7.5 × 10^−4^ *m* face sizing is used with a 8-node plane element (Plane-183), that was selected for its capacity to handle large deflections and support hyperelasticity.

In the DRL optimization, we used the reward function defined as

(7) $$\begin{array}{r} r=-\left(Y_{O_{1_{x}}}-Y_{O_{1_{y}}}\right)+\left(Y_{O_{2mid_{x}}}-Y_{O_{2mid_{y}}}\right)+\left(Y_{O_{3mid_{x}}}-Y_{O_{3mid_{y}}}\right), \end{array}$$

where *Y*_*O*_1_*j*___, *Y*_*O*_2_*j*___, and *Y*_*O*_3_*j*___, for *j* = *x, y*, correspond to the horizontal (*x*) and vertical (*y*) components of the respective nodes in the FE model, analogous to locations in the analytical model and corresponding reward function in (6). In each variant (4 actions and 8 actions), we use the same base reward function (7). In addition, we also considered the criteria of (6) for direct comparison. Both results are presented in the overall performance comparison of methods, however, the latter design was only numerically validated.

During the optimization process, given a random initial condition, each simulation was run for 500 time steps with a batch size of 16. The process was repeated until the final rewards repeatedly converge to the same maximal reward values to guarantee the optimal solution was found.

## 4. Results

### 4.1. Optimization Results Using Analytical Model

**Figure 4:**
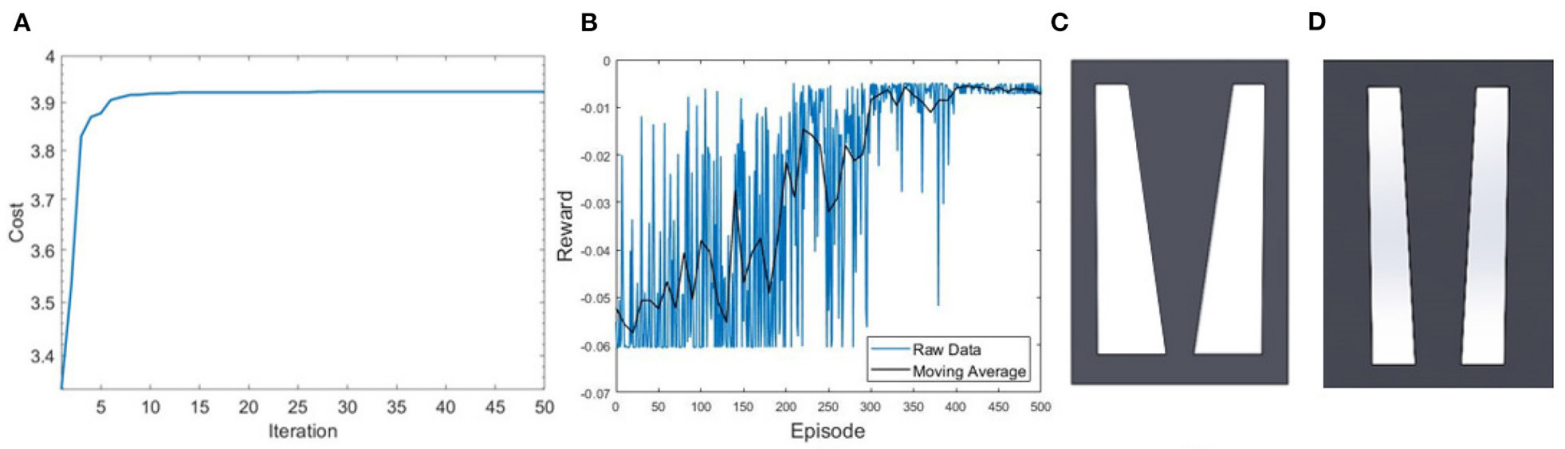
Performance of convergence toward optimal solution for **(A)** FA, where the best cost is achieved in 10 iterations, and **(B)** DDPG algorithms, where steady region was achieved after 400 episodes with 10 points included in the moving average. **(C)** Optimized design from the FA optimization with an estimated horizontal displacement of 0.015*m*. **(D)** Air chamber design from the DDPG based optimization at the steady state with model estimated displacement of 0.0103*m*.

The FA-based optimization exhibits fast convergence, reaching its steady value and best cost function in 10 iterations (see Figure 4A). DDPG-based optimization more gradually converges to an optimal reward value with large oscillations in the reward function, due to the DRL agent being updated in batches, and not directly considering the best cost in the process of the agent learning as is done in the FA approach.

Figure 4 shows a result comparison of optimal air chamber designs obtained from FA (Figure 4C) and DDPG optimizations (Figure 4D) both using the analytical model as the environment. The optimized model parameters of air chamber design using the FA and DDPG optimizations are (*b*_1_ = 0.0054*m*, $L_{1}^{\prime }=0.006m$, *b*_2_ = 0.025*m*, $L_{2}^{\prime }=0.0065m$) and (*b*_1_ = 0.010*m*, $L_{1}^{\prime }=0.011m$, *b*_2_ = 0.010*m*, $L_{2}^{\prime }=0.0083m$), respectively. Comparison of model estimated maximal horizontal displacements at the tip of the beams showed 0.0047 *m* greater displacement of FA (0.0150 *m*) compared to the DDPG (0.0103 *m*) optimization. Air chamber layout design comparison shows an increased size of the air chambers and reduced size of the side beams when using FA compared to the DDPG optimization. Resulting ratios of $b_{1}/L_{1}^{\prime }$ for the outer cantilevers are 0.9 for both FA and DDPG, reaching maximum allowed values. The ratio of $b_{2}/L_{2}^{\prime }$ of the central cantilever are 3.85 and 1.20 for FA and DDPG, respectively. Experimental and FE computational results of maximum displacements are presented later in this section.

### 4.2. DDPG With Finite Element Methods

**Figure 5:**
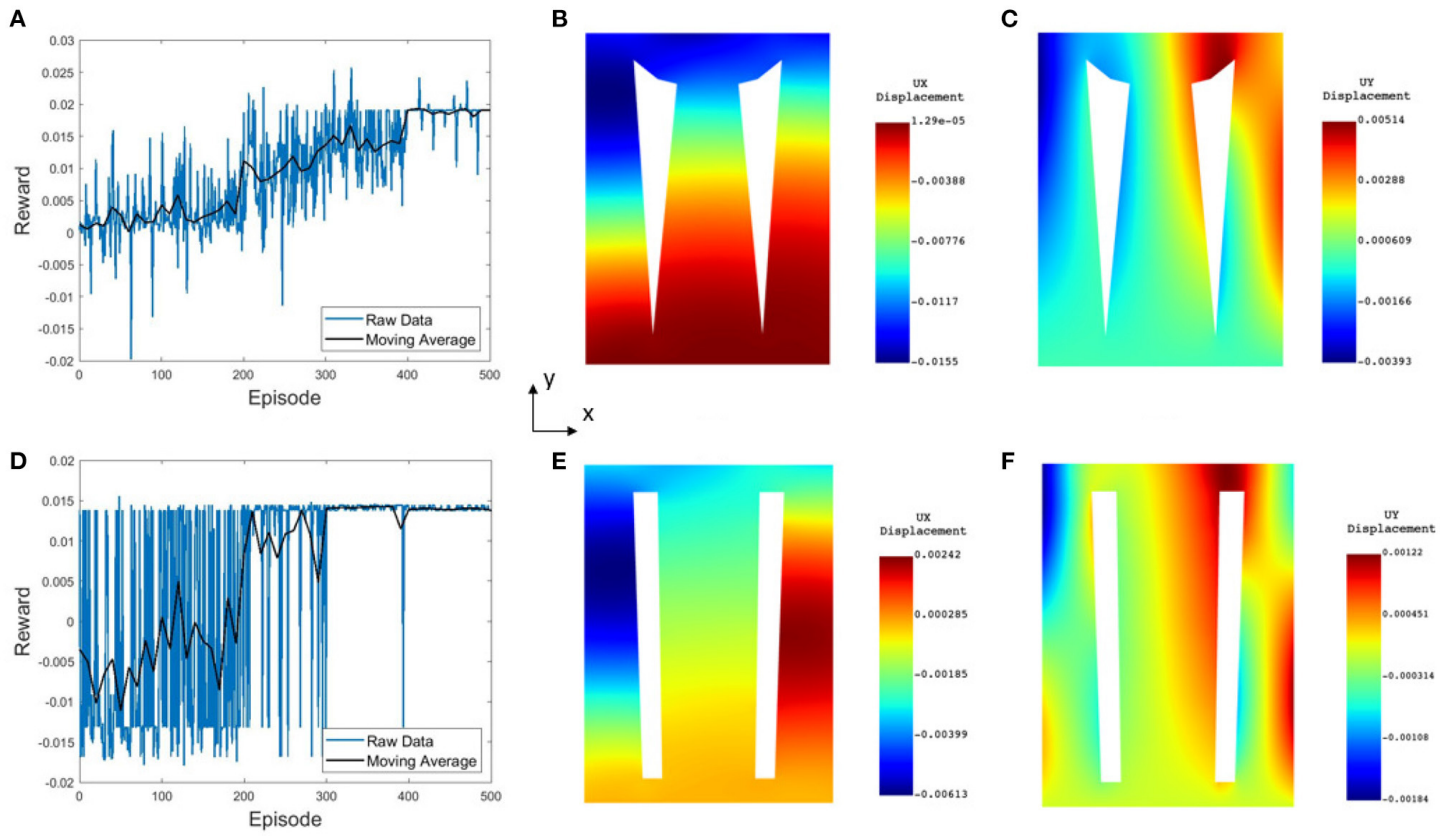
Numerical computation results from FE-based optimizations for **(A–C)** the 8 action FE model and **(D–F)** the 4 action FE model. **(A,D)** Convergence of the reward, where steady state is achieved in 400 and 300 episodes, respectively. **(B,E)** Horizontal displacement contour map, where the maximal displacement on the top surface approaches 15.5 and 6*mm*, respectively. **(C,F)** Vertical displacement contours.

Figure 5 shows the DDPG optimization results using the FE environment with unconstrained *x*− and *y*− coordinates having 8 actions (Figures 5A–C) and unconstrained *x*− and constrained *y*− coordinates having 4 actions (Figures 5D–F). In both optimized chamber geometries, the bottom of the chambers are significantly smaller than the results from the analytical model. The estimated displacement of designs from DDPG based optimization in Figure 5 are similar to those presented in Figure 4, which confirm the validity of the analytical model-based approach. Results from all simulations are further validated using a computational FE model and experimental validation later in this section.

### 4.3. Computational Model Results and Experimental Validation

The optimization results were validated using computational models and experimental testing (Figure 7). We built FE computational models in ANSYS and fabricated soft actuators from silicone rubber for both model-based designs, the 8-DoF FE DDPG optimization presented in Figure 5, and the empirically designed model from Raeisinezhad et al. (2020) with the modification of removing the top chamber for ease of results comparison. Additive manufacturing and casting techniques from Raeisinezhad et al. (2020) were used to create molds, soft actuators and material samples. In the experiments, the base of the actuator was fixed to a platform and a pressure differential was applied between the two side chambers. Pressure of 12 *psi* was applied in the right chamber and vacuum of −5 *psi* in the left. The full-field deformation response of each actuator is recorded and characterized using 3D digital image correlation (DIC). Free displacements of the top surface (i.e., edge point) of the actuators were analyzed and compared.

**Figure 6:**
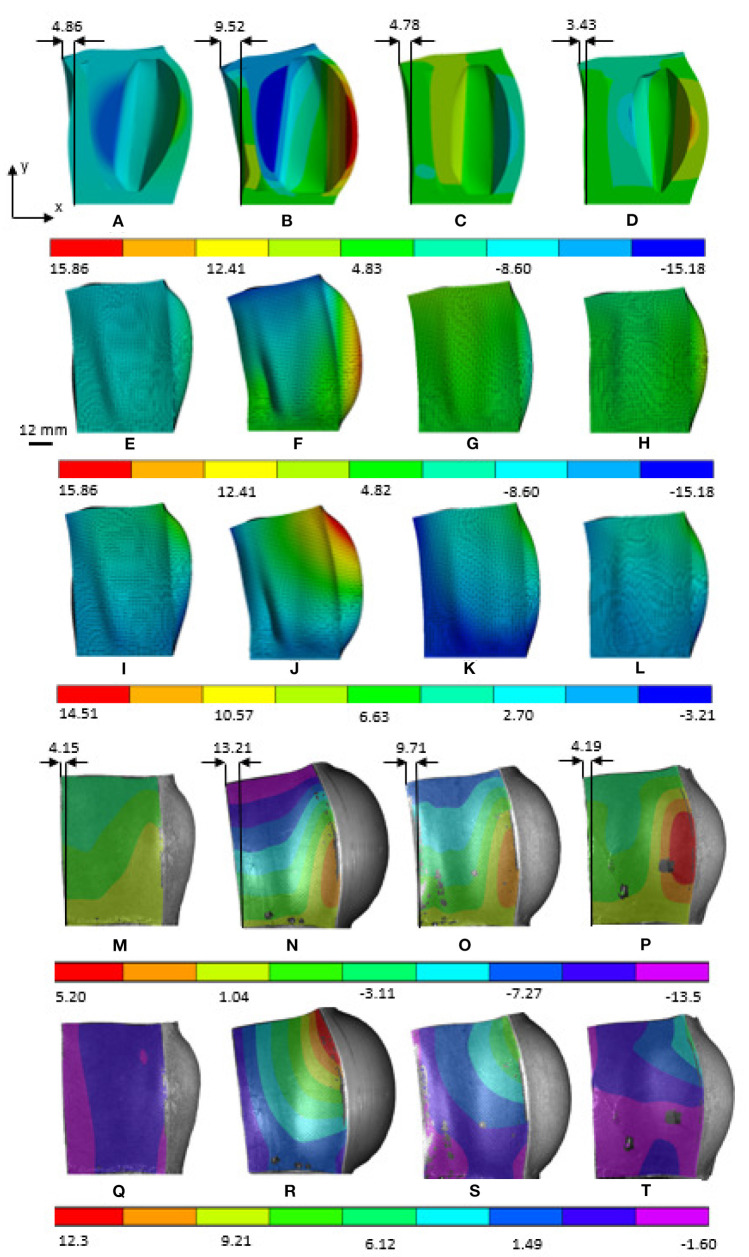
ANSYS and DIC experimental results of horizontal motion in x direction and vertical motion in the y direction as shown in the figure. **(A,E,I,M,Q)** Original dome, **(B,F,J,N,R)** Model-based FA, **(C,G,K,O,S)** Model-based DDPG, **(D,H,L,P,T)** 8-DoF FE-based.

Figure 6 shows a direct comparison of the FE computational model and DIC experimental results. Colors and contour bands correspond to the displacement field of each actuator with applied +12/−5 psi of pressure/vacuum simultaneously. Figures 6A–D show the horizontal motion at a center slice of each actuator to show the deformation of the beams internally. The most aggressive displacements are observed in the empirical and FA-based designs, while in all designs the cantilevers make contact. Comparison of the contour bands for horizontal displacements shows similar trends between the FE computation model (Figures 6E–H) and experimental results. Figures 6M–P shows matching trends with best match across the top surface in the original and FE-based designs. The vertical displacement results from FE computational model (Figures 6I–L) and experiments (Figures 6Q–T) show a matching displacement contours results with the increase in displacements in chamber with positive pressure across all designs.

**Figure 7:**
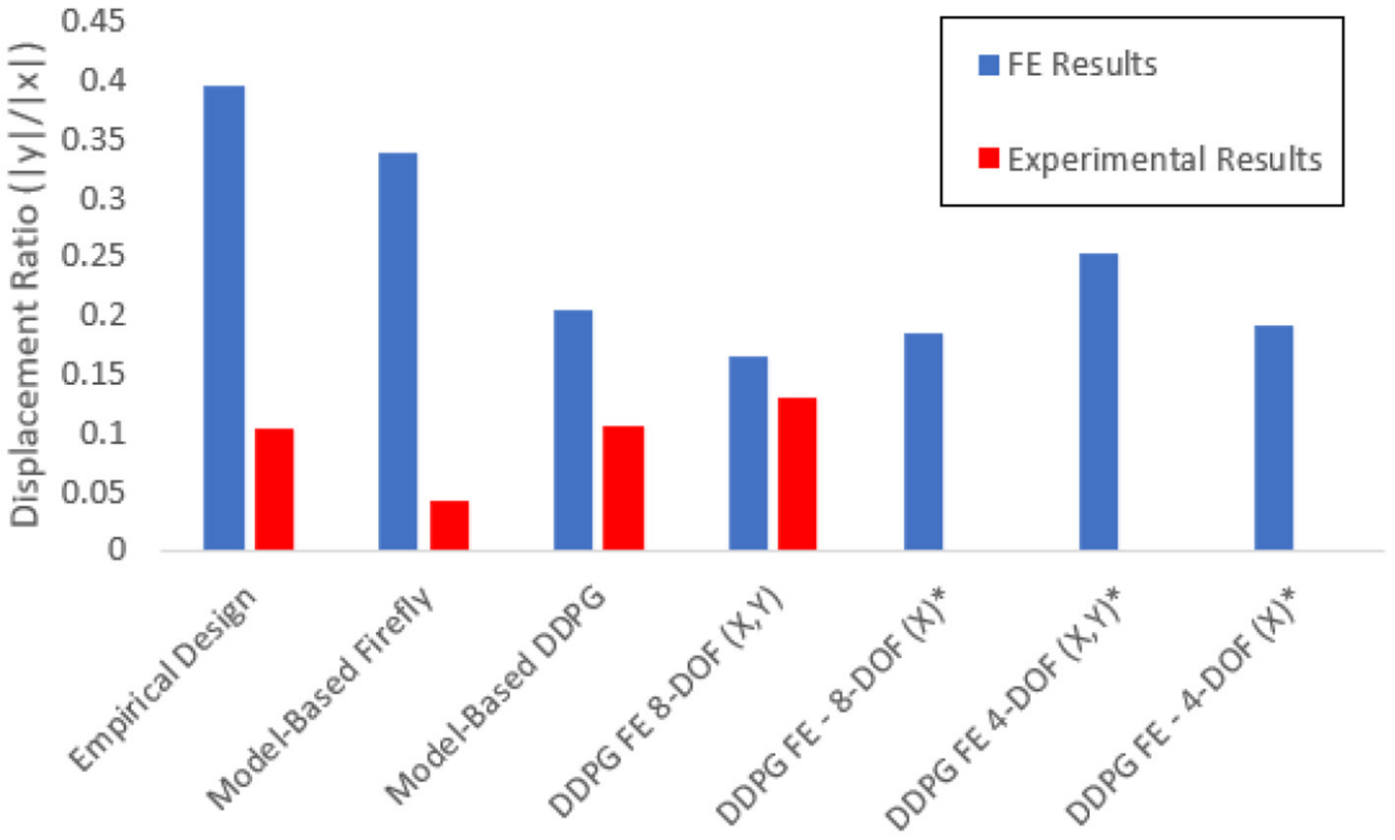
Comparison of the FE and experimental results for the ratio of maximum horizontal and vertical displacements for each design. Various cost function considerations are considered as discussed in the FE computational model for optimization section. Designs that were evaluated only computationally and were not manufactured are designated with *.

Due to our cost function considering the displacement of the first cantilever, we compare the tip displacements to evaluate the efficacy of the methods. Figure 7 shows the comparison of the experimental results of the ratios of maximal vertical and horizontal displacements (*Y*_*O*_1_*y*___/*Y*_*O*_1_*x*___) for all designs. We present the ratios to show decoupling of degrees of freedom at steady pressure conditions. A smaller ratio indicates better decoupling. All FA and DDPG-based optimized designs outperform the original empirical design from Raeisinezhad et al. (2020). Furthermore, all DDPG based designs have lower ratios than the FA validation method, indicating better performance. Experimental results show better decoupling than the FE models. We attribute this to the upward deflection present at the base of the actuators that is visible in Figures 6Q–T and was the most aggressive in FA designed actuator.

**Figure 8:**
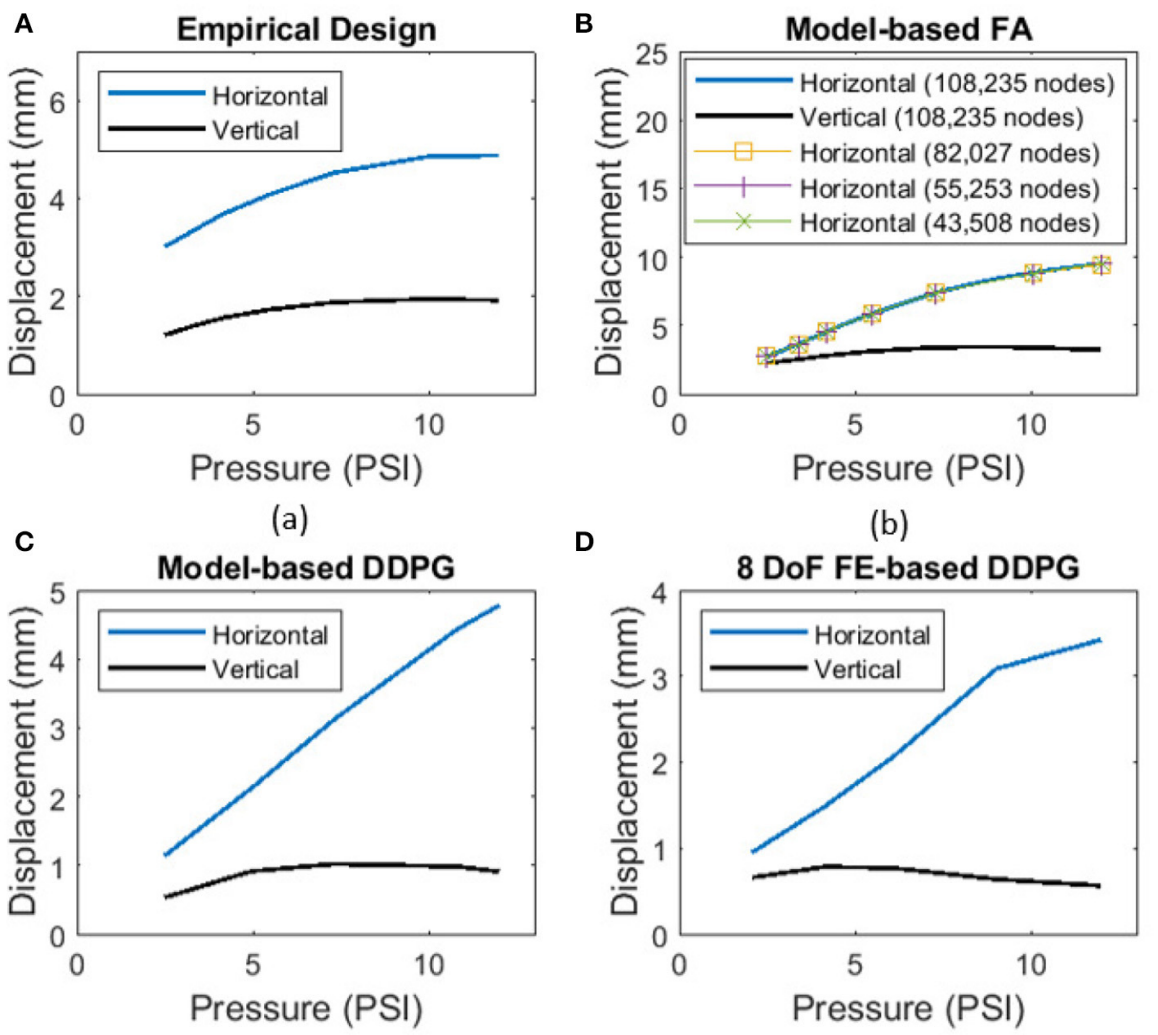
Relationship between horizontal displacement and applied pressure from FE-based computational models for all **(A)** empirical design, **(B)** model-based FA, **(C)** model-based DDPG, **(D)** 8-DoF FE-based DDPG optimization designs. Convergence is shown for nodes in model-based FA in **(B)**.

Figure 8 shows the relationship of the horizontal and vertical displacement based on the applied pressure/vacuum in air chambers for the path of displacement results presented in Figure 7. In addition, in Figure 8B, we show solution convergence by presenting the horizontal displacement for element sizes ranging from 1.5 mm (108,235 nodes) to 2.1 mm (43,508 nodes). This shows our mesh size is sufficient and does not effect the results, we use a 1.5 mm body sizing from this convergence. The results show near linear behavior in the horizontal direction for most of the domain. This is best shown in the DDPG-based designs (Figure 8). This extends into the validity of the proposed analytical model for this pressure range. The empirical design shows the least linear relationship. On the contrary, the vertical displacements all display a non-linear trend.

## 5. Discussion

**Figure 9:**
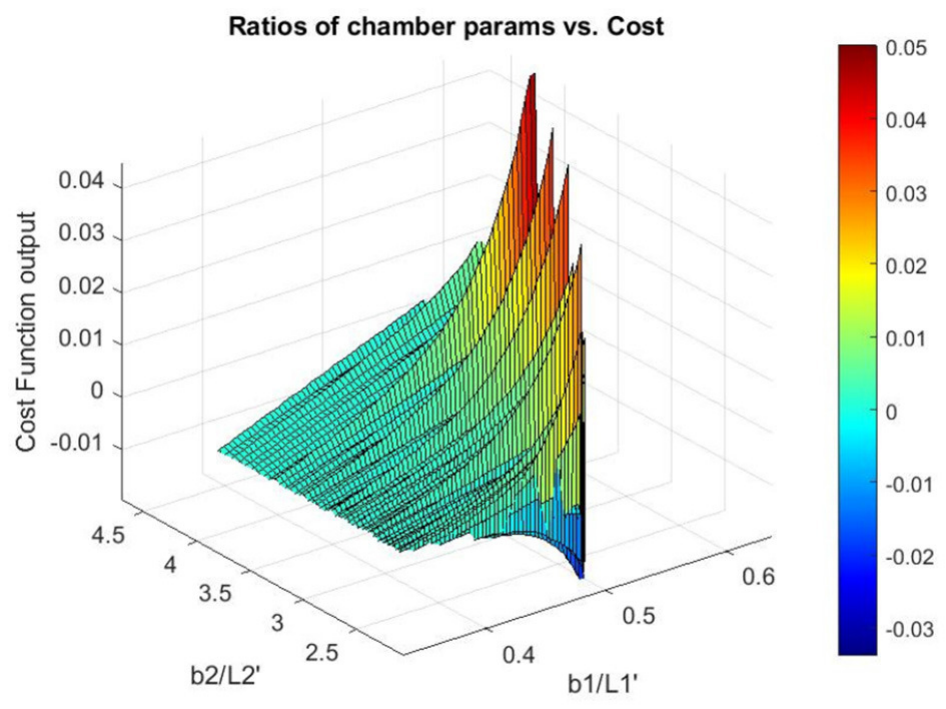
Surface of cost function with parameter ratios for the outer cantilever from Figures 1E,G, and the central cantilever from Figure 1F.

Figure 9 shows the ratios of input parameters for the inner and outer cantilevers compared to the output of the cost function. Only the cantilever from Figure 1 is shown for the outer, due to the symmetry assumption. Figure 9 shows that gradient-based methods may not be appropriate for this problem due to several minima and maxima. Graphically examining Figure 9 we find that the expected optimal design for the cantilever in Figures 1E,G approaches the ratio of *b*_1_ and $L_{1}^{\prime }$ being 0.65 or greater depending on the boundary dimensions. For beam in Figure 1F, *b*_2_ is much larger than $L_{2}^{\prime }$ with a ratio between 3.75 and 4.25. Due to large displacements from near zero α_*i*_ values, geometries that approach rectangular chambers can also be anticipated for the model-based approach.

We acknowledge that the solution of the elementary beam theory used as our analytical model is not valid for large beam deflections, due to neglecting the square term of the first derivative (slope deflection) in the curvature formulation. Based on the comparison results among the elementary and large deflection beam theories in Bisshopp and Drucker (1945), we propose that our modeling approach could be used for the beam deflection to length ratio of up to approximately 0.3 to yield a reasonable solution. The results in Figure 8 show that the maximum beam deflection to length ratio (*y*_*i*_/*L*_*i*_, for *i* = 1, 2, 3) at maximum applied pressure/vacuum across all designs does not exceed 0.15, which validates use of the proposed approach. Figure 8 also shows that the pressure to horizontal displacement relationship is nearly linear for the considered pressure range and we conclude that our structural modeling approach is suitable to adequately capture the horizontal displacement in our application. In addition, although the utilized material is categorized as a non-linear elastic one, the degree of its non-linearity is insignificant at least up to strains of 0.4 (see Figure 2). The range of strains considered in this work (determined by FEM and DIC measurements, Figure 6) allow us to assume that the material's phenomenological response can be assumed nearly linear up to strains of about 0.4, at least for optimization purposes.

The development of systematic modeling and optimization tools for soft actuator design lead to improvements of its functionality in the horizontal motions that the actuator can achieve. Considering horizontal displacements of the design optimized using the FA method (Figure 4C) showed a 4.63 *mm* improvement in the experiments, and the DDPG variant showed a 4.78 *mm* deflection which is 0.09 *mm* less. However, the vertical displacements show a 1.01 *mm* improvement over the empirical design, which the FA design was not able to capture. Examining the ratio of vertical to horizontal motion model-based DDPG was able to nearly half the ratio of the empirical design from 0.39 to 0.20. Since the formulations are based on a 2-D model, the model cannot fully capture pure planar horizontal motion and the actuators exhibit some bending motions. However, from Figure 8 the near linear behavior after initial input leads us to assume that the equations are valid for the entire range, and the optimal solution can be realized with this model. Further decoupling of the degrees of freedom can be achieved by deeper consideration into the vertical dynamics of the actuator and improving the analytical model to replace cantilever beams with constrained beams. FE based designs all show close results for displacement ratios, implying that our cost function is most dependent on the horizontal displacements for effective use. We choose to manufacture the 8 DOF, x,y variant since it had the lowest displacement ratio in Figure 7. Possible future considerations may include addition of a term to minimize the bubbling effect seen in Figure 6 on each design.

Observing the designs, a larger chamber size and thinner side walls of the soft actuator result in larger outward expansion and lower mechanical stiffness in the vertical direction. This is at the penalty of increased bending motion. The contradicting objectives demonstrate the need for a design trade-off between maximizing horizontal motion and minimizing vertical motion. Reduced axial mechanical stiffness leads to smaller weight bearing capabilities and possible buckling when a normal load is applied on top of the actuator. The weight bearing capabilities are important when using the soft actuator in pressure and blister injury prevention applications and must be further validated.

In this work, DDPG outperformed the FA based validation method, and was exclusively selected for implementation with a FE computational model. The validation method was selected due to its documented efficacy in being able to handle global optimization problems of assorted types and to encourage general use in soft robotics problems where non-convexity may cause gradient methods get stuck in a local minima or maxima. We note that in our optimization methods for both model-based and FE-based environments, we run DDPG multiple times and compare steady values of the reward signals, which is primarily because the method is sensitive to starting conditions. This has been documented in Xu et al. (2018) which explores meta-policy gradient methods due to problems in noise-exclusive exploration methods that can limit the region that the policy explores. In addition, DDPG itself is very sensitive to parameters, which should be considered for further use of this particular DRL-based method. The abundance of open-source DRL codes (Hill et al., 2018) can provide many avenues of future exploration of DRL for design optimization with proper objective formulation to address some of these issues. Due to our focus being primarily on the DRL-based optimization, we did not consider the FA-based optimization combined with the FE model that was out of the scope of this paper. This is the limitation of this work and considering this combination is one among our future research directions.

## 6. Conclusions

In this paper, we present the design optimization of a novel soft robotic actuator for surface manipulation applications. We extend the analytical formulations for tapered and thickened beams that are connected with spring elements to create a model of a multi-chamber actuator with a single degree of freedom displacement. While simple in nature this model captures nominal displacement of the geometries well for the considered pressure domain with near linear behavior as validated through FE computational model.

DDPG-based optimization and a firefly validation algorithm were used in the design optimization process for the developed analytical model. DDPG optimization was extended to use a finite element model as its environment. Computational models of the optimized actuator designs were created and analyzed using a commercial FE solver to validate their mechanical performance. Actuators with notable degree of freedom decoupling were fabricated and the full-field deformation was characterized using digital image correlation. All FA and DDPG-based designs decouple motion better than the empirical design. FA produced the largest horizontal motion but also exhibited the largest vertical deflection. Model-based DDPG performed nearly equal to the empirical design in horizontal motion with notable improvement in vertical motion reduction in simulation, and outperformed the empirical design in simulation. The selected FE-based DDPG design yields the best decoupling ratio, with lower displacements. The improved decoupling of motion and appropriate horizontal displacements of the FE-based DDPG design makes this design suitable for pressure injury and blister prevention. Our future work includes continued design optimization and control of a soft multi-actuator surface manipulation system used in controlled human-machine interaction applications.

## Data Availability Statement

The raw data supporting the conclusions of this article will be made available by the authors, without undue reservation.

## Author Contributions

MT designed the study and directed the project. MR and MT developed the theoretical framework. MR and NP performed the experiments and simulations. MR, NP, BK, and MT analyzed the data, interpreted and discussed results, and wrote the paper with input from all authors.

## Conflict of Interest

The authors declare that the research was conducted in the absence of any commercial or financial relationships that could be construed as a potential conflict of interest.
